# Differential outcomes of novel plant-herbivore associations between an invading planthopper and native and invasive *Spartina* cordgrass species

**DOI:** 10.1007/s00442-021-04898-8

**Published:** 2021-03-31

**Authors:** Claire Harkin, Alan J. A. Stewart

**Affiliations:** School of Life Sciences, University of Sussex, Brighton, BN1 9QG UK

**Keywords:** *Prokelisia marginata*, Biological invasions, Plant–insect interactions, Polyploidy, Enemy release

## Abstract

Non-native plants may benefit, briefly or permanently, from natural enemy release in their invaded range, or may form novel interactions with native enemy species. Likewise, newly arrived herbivores may develop novel associations with native plants or, where their hosts have arrived ahead of them, re-establish interactions that existed previously in their ancestral ranges. Predicting outcomes from this diversity of novel and re-established interactions between plants and their herbivores presents a major challenge for invasion biology. We report on interactions between the recently arrived invasive planthopper *Prokelisia marginata*, and the multi-ploidy *Spartina* complex of four native and introduced species in Britain, each representing a different level of shared evolutionary history with the herbivore. As predicted, *S. alterniflora*, the ancestral host, was least impacted by planthopper herbivory, with the previously unexposed native *S. maritima*, a nationally threatened species, suffering the greatest impacts on leaf length gain, new leaf growth and relative water content. Contrary to expectations, glasshouse trials showed *P. marginata* to preferentially oviposit on the invasive allododecaploid *S. anglica*, on which it achieved earlier egg hatch, faster nymphal development, larger female body size and greatest final population size. We suggest *P. marginata* is in the process of rapid adaptation to maximise its performance on what is now the most abundant and widespread host in Britain. The diversity of novel and re-established interactions of the herbivore with this multi-ploidy complex makes this a highly valuable system for the study of the evolutionary ecology of plant–insect interactions and their influence on invasion dynamics.

## Introduction

Biological invasions are recognised as one of the primary drivers of biodiversity loss, responsible for significant ecological and economic costs worldwide (Mack et al. 2000; IPBES 2019). Most ecological communities now contain at least one non-native species, with invaders already representing over a fifth of many countries’ flora (Mooney and Cleland 2001). At least 13,168 species of vascular plant are known to have become naturalised outside their native range, with almost 5000 of them causing harm to the environment, the economy or human health (RBG Kew 2016).

A popular explanation for the success of invasive plants is that they often arrive in their new range without the full suite of natural enemies (herbivores, fungi and other pathogens) with which they have co-evolved (Maron and Vila 2001; Keane and Crawley 2002). Newly arrived plants may benefit from such natural enemy release, briefly or permanently, or they may form novel interactions with native enemy species in the new range. Likewise, newly arrived herbivores may develop novel associations with native plants or, where their hosts have arrived ahead of them, re-establish interactions that existed previously in their ancestral ranges. Predicting the outcomes from such a diversity of novel and re-established interactions between plants and their herbivores presents a major challenge for invasion biology (Chun et al. 2010; Pearse et al. 2013; Bezemer et al. 2014; deJonge et al. 2019).

One frequently encountered circumstance of particular interest concerns where a non-native introduced plant is reunited with its herbivore after temporarily benefitting from natural enemy escape in its new range. This may happen because the herbivore arrives naturally or anthropogenically, the latter being either accidental or as a deliberate attempt at biocontrol. The strength and character of the re-established association between plant and herbivore may differ from that found in their native range due to the influence of a suite of biotic and abiotic factors (Mitchell et al. 2006). As the plant and herbivore share a long evolutionary history prior to their introduction into the new range, the prediction is that the plant in its non-native environment will be less severely affected by the herbivore than native congenerics with no prior exposure. Indeed, some studies show reassociation with historic enemies can result in levels of plant defence greater than those displayed in their shared native range (Zangerl and Berenbaum 2005; Lu and Ding 2012), although evidence that greater defence results in greater comparative performance remains equivocal (Chun et al. 2010). Due to the greater length of shared evolutionary time in which reciprocal adaptations have been able to develop, the herbivore is predicted to perform better on its coevolved host compared to on congeneric natives. Coevolved hosts have been shown to support a greater abundance and diversity of insect herbivores, with significant host discrimination by phloem-feeding insects persisting despite the assumed palatability of novel alternatives (Burghardt and Tallamy 2013). Such preferences are frequently correlated with greater performance outcomes (Gripenberg et al. 2010).

A further complication may arise if ploidy levels differ amongst sympatric congeneric species. Polyploidy is widespread in plants (Ramsey and Schemske 1998), especially grasses (Stebbins 1956), and in particular occurs at elevated frequencies amongst invasive plants compared to angiosperms in general (Prentis et al. 2008; Pandit et al. 2011; te Beest et al. 2012). Fitness differences between populations with different ploidy levels have been reported in some species complexes (Soltis and Soltis 2000; Prentis et al. 2008; Pandit et al. 2014), but the effects of polyploidy on plant-animal interactions remains relatively unexplored (Thompson et al. 2004; Munzbergova 2006; Munzbergova et al. 2015). Ploidy has been shown to affect the level of damage that herbivores exert on conspecifics (Lou and Baldwin 2003). In many systems, higher-level cytotypes are subject to increased attack, however this is not universal and the preferred ploidy level can differ between even very closely-related herbivore species (Munzbergova 2006; Halverson et al. 2008; Segraves and Anneberg 2016). Reciprocal effects may also be evident, with consequent impacts on herbivore success resulting in further uncertainty in predicting the outcome of biological invasions mediated by plant–herbivore interactions (Hull-Sanders et al. 2009). This may necessitate system-by-system investigations to inform management interventions.

The introduction of the cordgrass *Spartina alterniflora* Loiseleur to Britain from North America, its hybridization with a threatened native congeneric species, including a chromosomal doubling event, and subsequent re-connection in the new range with its ancestral herbivore, the planthopper *Prokelisia marginata* Van Duzee, provides a unique opportunity to test these predictions. Here, we compare the impact of exposure to *P. marginata* on four species of *Spartina* in Britain with different histories of co-occurrence with the herbivore: the introduced ancestral host *S. alterniflora*, the native and previously unexposed *S. maritima*, the homoploid hybrid of these two species, *S. x townsendii,* and the allododecaploid *S. anglica* which arose from a chromosomal doubling of *S. x townsendii*. Also, we investigate whether *P. marginata* makes a preferential choice between the host species, and the impact that these host species have on *P. marginata* performance and fitness outcomes. We hypothesise that: (1) *P. marginata* will preferentially select *S. alterniflora*, the species with which it has the longest shared evolutionary history, for feeding and oviposition; (2) host plant species will have a significant impact on *P. marginata* life history traits, with the planthopper achieving the greatest performance outcomes when raised on species with which it has the longest shared evolutionary history; (3) exposure to *P. marginata* feeding and oviposition will have a deleterious impact on all species of *Spartina*, but the severity of impact will be related to the extent of shared evolutionary history, i.e. least for *S. alterniflora* and greatest for *S. maritima*; and (4) the level of impact will not differ significantly between *S. x townsendii* and *S. anglica,* despite their differing ploidy levels, because they share identical evolutionary histories with the planthopper and because *S. anglica* originated from a chromosomal doubling of *S. x townsendii* without the introduction of additional genetic material.

## Materials and methods

### Study system

*Spartina* is a genus of perennial rhizomatous polyploid C_4_ grasses containing around fifteen species, including a number of hybrids (Ainouche et al. 2009; Strong and Ayres 2013; Bortolus et al. 2019). Most *Spartina* species are primary colonists of intertidal mud flats and have been intentionally introduced to many parts of the world due to their ability to trap sediment and thereby stabilise eroding shorelines, reclaim land and provide defence against extreme coastal weather events (Callaway and Josselyn 1992).

**Fig. 1:**
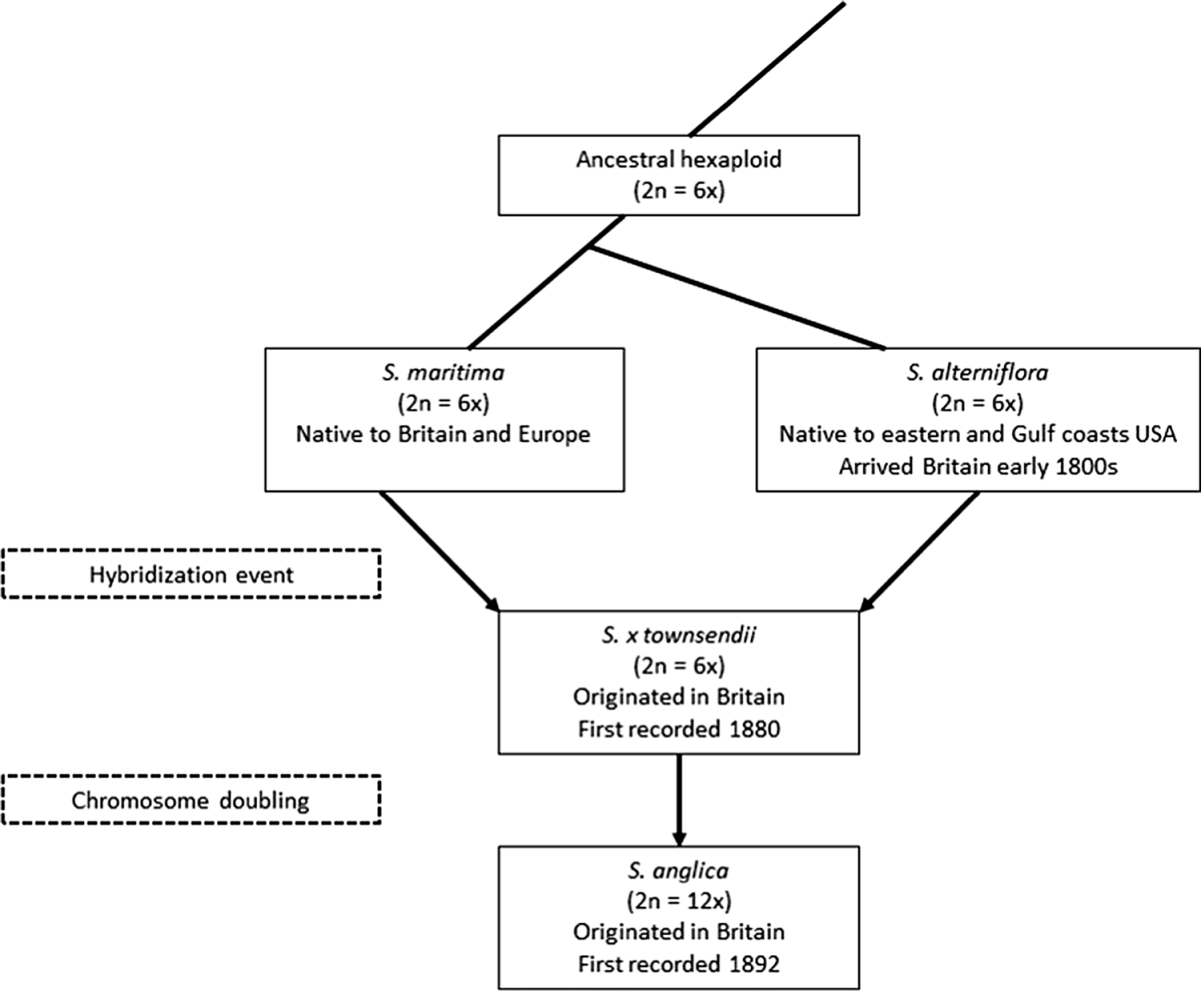
Geographical origin, ploidy levels, hybridization and allopolyploid speciation of *Spartina* species in Britain following *S. alterniflora* introduction

Four species of *Spartina* are present in Britain, only one of which, *S. maritima* (Curtis) Fernald, is ancestrally native. Populations of the non-native *S. alterniflora* are presumed to have been established from seeds transported in shipping ballast from the eastern coast of North America in the early nineteenth century (Thompson 1991). Interspecific hybridization of *S. alterniflora* with *S. maritima* resulted in the sterile homoploid hybrid *Spartina x townsendii* Groves, first described in 1880 from samples collected at Hythe, Hampshire on the south coast of England (Groves and Groves 1880). Fertile plants were first recorded in nearby Lymington in 1892 which appeared to have resulted from chromosome doubling in *S. x townsendii* (Marchant 1967) and were later described as the new fertile allododecaploid species *S. anglica* Hubbard (Hubbard et al. 1968) (Fig. 1). *S. anglica* rapidly colonised British coasts through natural dispersal both of seeds and rhizomes and by deliberate introduction for saltmarsh reclamation (Thompson 1991), and is now the dominant structuring species in a quarter of Britain’s lower saltmarsh communities (Gray et al. 1997). The three progenitor species all still occur in extremely small and localised populations, but *S. maritima* is listed as a species “of principal importance for the purpose of conserving biodiversity” under section 41 of the UK Natural Environment and Rural Communities Act 2006 and a priority species under the UK Biodiversity Action Plan (Joint Nature Conservation Committee 2007). Understanding the potential impacts of a newly arrived specialist herbivore is therefore of great importance for the conservation management of this species.

The planthopper *P. marginata* is native to the Gulf and Atlantic coasts of North America where it is the most abundant herbivore of *S. alterniflora*, frequently reaching densities exceeding 1000 adults/m^2^ and 100,000 nymphs/m^2^ (Denno et al. 1986). Despite their high densities, *P. marginata* have been found to have only weakly adverse effects on *S. alterniflora* where the native ranges of both species coincide (Gustafson et al. 2006; Roberts and Pullin 2008) as well as in invaded ranges where both have coexisted for several decades (Daehler and Strong 1995). However, *S. alterniflora* populations lacking a recent history of co-occurrence with *P. marginata* suffered significant reductions in growth and survival when re-exposed to the herbivore (Daehler and Strong 1997; Wu et al. 1999; Garcia-Rossi et al. 2003).

Across its North American range, *P. marginata* has been reported to feed only on *S. alterniflora*, *S. foliosa* and their hybrids, including the introduced *S. anglica,* whilst avoiding the sympatrically-occurring *S. patens*, *S. cynosuroides* and *S. bakeri* (Denno et al. 1996), the latter occupying a more distantly related clade (Baumel et al. 2002b). No-choice host specificity tests showed that *P. marginata* consistently achieved the greatest survival rates on *S. alterniflora* compared to the other three *Spartina* hosts and was unable to complete a full life cycle on any of twenty other plant species, including other *Spartina* spp., closely- and distantly-related monocotyledons and one dicotyledonous species (Grevstad et al. 2003). Nymphs raised on more nutritious host plants eclose as significantly larger adults, which in females is positively correlated with daily fecundity. In contrast, high population density has been shown to reduce survivorship, decrease body size and delay nymphal development, consequently increasing the age of first reproduction and reducing lifetime realised fecundity (Denno and McCloud 1985). Nymphal emergence, development and survivorship are negatively impacted by poor quality or unsuitable hosts (Garcia-Rossi et al. 2003; Grevstad et al. 2003), whilst highly nutritious hosts moderate the fitness-reducing impacts of crowding (Denno et al. 1986).

*P. marginata* is presumed to have arrived in Britain sometime since 2000, with preliminary studies suggesting it is in the early stages of successful invasion (Harkin and Stewart 2020). Across its introduced continental European range, *P. marginata* has been recorded primarily on *S. anglica* or *S. x townsendii* (de Blauwe 2011), with a single Slovenian population recorded on *S. maritima* (Seljak 2004). Prior to the work presented here, *S. anglica* was the only recorded host for *P. marginata* in its British range (Badmin 2013; Harkin and Stewart 2020).

### *Spartina* spp. and *Prokelisia marginata* experimental material

*Spartina* spp. source populations were identified at four sites along the south coast of England: *S. alterniflora*—Hythe (50^o^86′N, 1^o^39′W); *S. anglica*—Pagham (50^o^77′N, 0^o^78′W); *S. maritima*—Hayling Island (50^o^83′N, 0^o^97′W); and *S. x townsendii*—Beaulieu Estate (50^o^77′N, 1^o^40′W). Due to the extremely sparse and localised distribution of all populations apart from *S. anglica*, it was not possible to collect sufficient quantities of more than one species from the same site. *Spartina* spp*.* plants were grown from sampled rhizome material that had been washed, cut to approximately 12 cm lengths including at least one node and planted in 10 cm (then later transferred to 15 cm) diameter pots containing horticultural grade silver sand. Pots were watered with fresh water and kept continually wet but not inundated (following Denno et al. 2000), with the addition of 100% Hoagland nutrient solution (Hoagland and Arnon 1950) fortnightly. Plants were grown under glasshouse conditions with supplementary lighting (100 W Supanova LED grow lights, 8:2 light ratio comprising 660 nm Red and 430 nm Blue) on an 18:6 h light:dark cycle. Plants were acclimated to glasshouse conditions for 16 weeks prior to the start of experiments.

*P. marginata* individuals used in glasshouse experiments were drawn from a breeding culture maintained on clusters of potted *S. anglica* plants grown under glasshouse conditions. The culture was initiated using *S. anglica* plants removed from Hythe showing brown markings indicative of *P. marginata* oviposition. New plants were added to the culture as required to maintain a consistent supply of host plant material. Second generation glasshouse-reared insects were utilised for the experiments.

### *P. marginata* oviposition choice in *Spartina* sp. field assay

Ten potted plants of each of the four *Spartina* species were transported to the *Spartina*-dominated marsh at Hythe, a site previously shown to have an established *P. marginata* population (Harkin and Stewart 2020). Hythe is considered to be the site of origin for both *Spartina x townsendii* and *Spartina anglica* (Raybould et al. 1991), although it is no longer possible to locate the former at the site. *S. alterniflora* is still present in a monospecific stand of approximately 125m^2^, with the remainder of the marsh populated by *S. anglica* (Renny-Byfield et al. 2010). Experimental plants were arranged in ten groups, with each group containing one individual of each species. Groups were randomly distributed in an area of established saltmarsh dominated by *S. anglica* measuring 20 m × 30 m, with a minimum of 1.5 m between each group. Within each group, plants were maintained in separate pots, arranged 10 cm apart in a 2 × 2 grid. Each group of pots was buried so that the tops were level with the surrounding substrate. After 24 days, all leaf material of the experimental plants was removed, measured for leaf length and examined under a dissecting microscope for *P. marginata* eggs. *P. marginata* egg density in each plant was expressed as the number per cm of combined lengths of all leaves.

### *P. marginata* oviposition choice between *Spartina* sp. under glasshouse conditions

Ten plants of each *Spartina* species were randomly assigned to one of ten groups, each containing a single plant of each species rooted in separate pots. Each group was enclosed in a cylindrical PET polyester cage as before, and eight female and four male adult *P. marginata* were introduced to the centre of each cage. After 14 days, leaf measurements and a count of *P. marginata* eggs were used to calculate the number of eggs per cm of combined leaf length for each *Spartina* species.

### Impact of *Spartina* sp. on *P. marginata* development times under glasshouse conditions

Two male and two female newly-emerged adult *P. marginata* were caged on each of twelve plants of each of the four *Spartina* species. Cages were monitored for 64 days to determine the date of first egg hatch, the date of first adult emergence and the date by which all adults had emerged. At the end of the experimental period, all *P. marginata* remaining in each cage were counted and the above-ground material of all plants was harvested by cutting at ground level, dried and weighed.

### Impact of *Spartina* sp. and crowding densities on *P. marginata* body size under glasshouse conditions

For each of the four *Spartina* species, five caged plants were randomly assigned to each of three planthopper crowding treatment levels: inoculation with ten (low), thirty (medium) or fifty (high) first-instar *P. marginata* nymphs respectively. After 64 days, above-ground plant material was harvested, dried and weighed, and the body size of all *P. marginata* adults was measured as the distance from the anterior margin of the head to the tip of the abdomen.

### *P. marginata* impact on *Spartina* species

Twenty plants of each *Spartina* species were randomly assigned in equal numbers to ‘herbivore’ and ‘control’ treatments. The following starting metrics were recorded for each plant: number of leaves; overall height; length of each leaf. Each plant was enclosed by a transparent cylindrical cage constructed from 175 µm PET polyester film, 13 cm in diameter, 50 cm tall and with a nylon gauze lid and a 5 × 7 cm gauze-covered ventilation window positioned 18 cm above the base of the cage. Replicates allocated to the herbivore treatment were inoculated with 30 s instar *P. marginata* nymphs, whilst those in the control group were maintained free of planthoppers. Individual plants were arranged in a randomized block design and maintained within a glasshouse for 8 weeks. At the end of the experimental period, all *P. marginata* adults and nymphs were individually removed and counted. Repeat metrics were recorded for each plant. Plants were then weighed to an accuracy of 0.01 g using a Precisa 125A balance, dried for 72 h at 70 °C in a Gallenkamp OV-420 drying oven and finally re-weighed to establish relative water content.

### Statistical analyses

Statistical analyses were performed with R version 3.1.3 (R Core Team 2015) using the *nlme* (Pinheiro et al. 2012), *lme4* (Bates et al. 2012), *effects* (Fox 2003) and *multcomp* (Hothorn et al. 2008) packages. Appropriate models were determined by the nature of the response variables. For the impact of *P. marginata* exposure on continuous plant measurements, ANOVA was used with change in the plant metric as the response variable and treatment, species and their interaction as explanatory variables. Oviposition choice data were analysed using a linear-mixed effect model (LMM). Other data were analysed with GLMMs using a Poisson distribution for count data and a binomial distribution for binary response variables. Each analysis began by fitting all relevant explanatory variables, interactions and random factors (block or plant IDs) in a maximal model. Model simplification then proceeded by a backwards deletion of non-significant terms until further removals led to a significant (*p* < 0.05) increase in deviance. This was assessed by comparing the model with and without the term in question using log-likelihood ratio tests for LMMs and *χ*^2^ values for GLMMs. Plots of model residuals against fitted values were visually inspected for normal distribution, homogeneity of variance and the presence of influential outliers. Results showing significant treatment effects were further investigated using Tukey HSD (Honest Significant Differences) *post-hoc* tests to identify differences between treatment means.

## Results

**Fig. 2:**
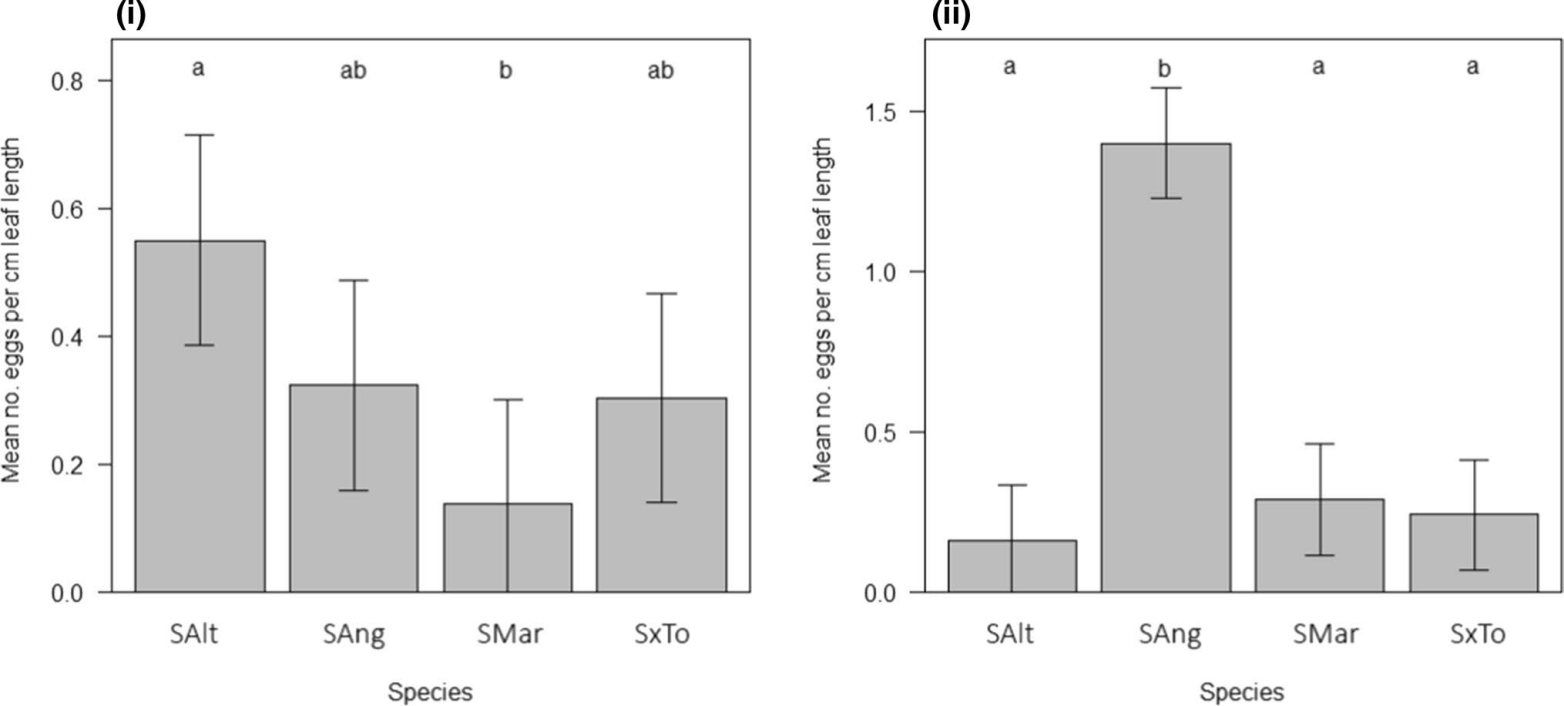
Mean number of *P. marginata* eggs laid per cm of *Spartina* spp. leaf length in **i** field and **ii** glasshouse experiments. Species abbreviations refer to: *S. alterniflora* (SAlt), *S. anglica* (SAng), *S. maritima* (SMar) and *S. x townsendii* (SxTo). Treatments sharing lower case letters are not significantly different from each other (Tukey HSD post-hoc test, using 95% CI). Error bars show means ± 1 S.E.M

Oviposition rates varied significantly among host plant species in both the field (*L* = 7.87, *p* = 0.049) and glasshouse (*L* = 26.85, *p* < 0.001) experiments (Fig. 2). Under field conditions, 75% fewer eggs were laid on *S. maritima* than *S. alterniflora*, but there were no significant differences in any other two-species comparisons. In the glasshouse experiment, *P. marginata* laid significantly more eggs in *S. anglica* compared to all other species.

**Fig. 3:**
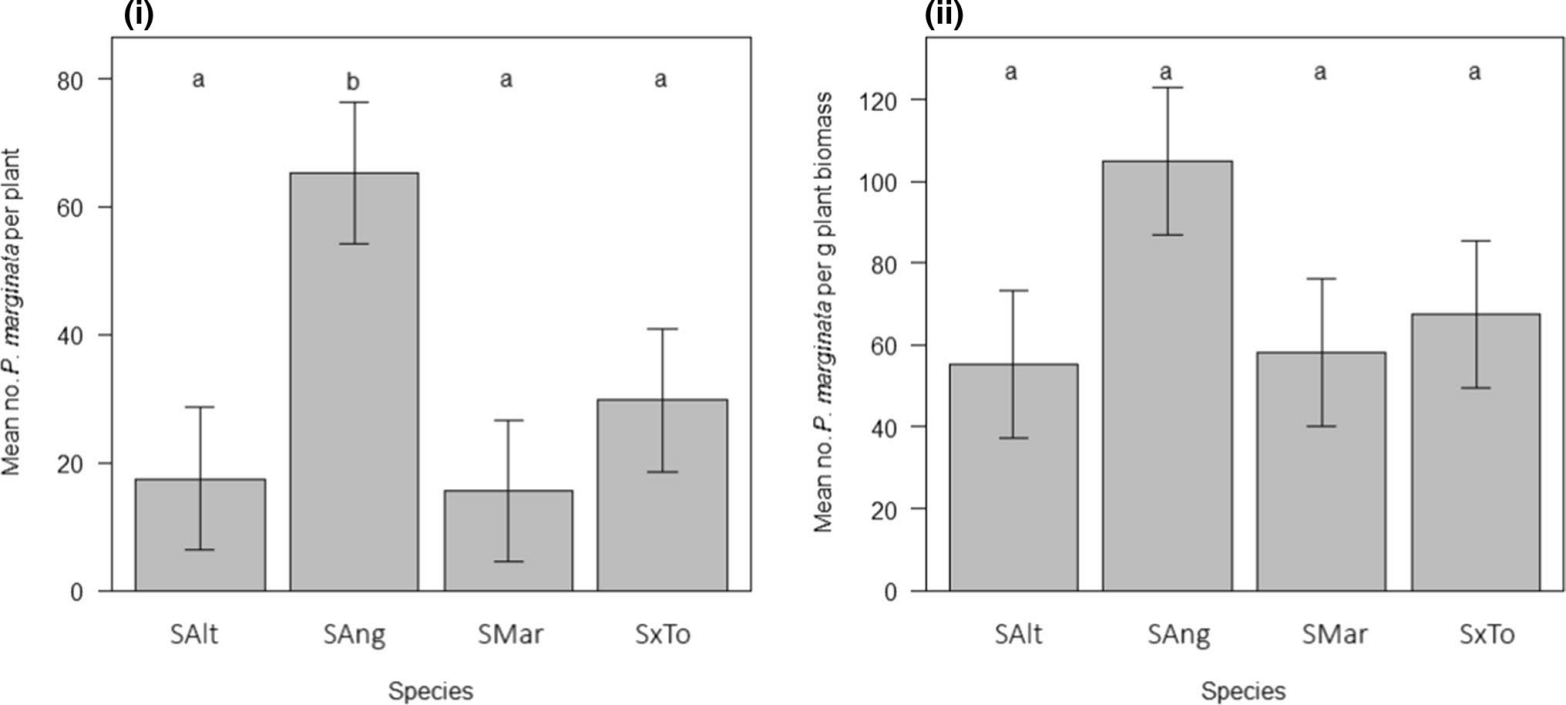
Mean number of *P. marginata* **i** per plant and **ii** per gram of *Spartina* dry biomass reared from four species of *Spartina* under glasshouse conditions. Species abbreviations as per Fig. 1. Error bars show means ± 1 S.E.M. Treatments sharing lower case letters are not significantly different from each other (Tukey HSD post-hoc test, using 95% CI)

In glasshouse choice experiments, plant species had a significant effect on the final number of *P. marginata* adults per plant (*L* = 15.58, *p* = 0.001), with the mean number of individuals on *S. anglica* between two and five times greater than on any other species. The mean number of *P. marginata* per gram of dry *Spartina* biomass was also much greater on *S. anglica*, although the overall difference between host plant species was not statistically significant (*L* = 5.26, *p* = 0.153) (Fig. 3).

There was a significant effect of host plant species on mean time to first adult emergence (*χ*^2^ = 8.29, *p* = 0.04), which was at least 3 days shorter for individuals reared on *S. anglica* compared to those on *S. alterniflora* or *S. maritima.* Time to first egg hatch was also shortest for eggs laid on *S. anglica*, although the overall effect of host plant species was not significant (*χ*^2^ = 1.37, *p* = 0.714) (Table 1).

**Table 1:** Effect of host plant species on the number of days to first *P. marginata* egg hatch and to first adult emergence

	d.f	Mean (SEM)	*χ*^2^	*p*
Days to first egg hatch	7		1.37	0.714
Species				
*S. alterniflora*		16.83 (1.40)		
*S. anglica*		15.00 (0.30)		
*S. maritime*		15.90 (0.77)		
*S. x townsendii*		16.33 (1.04)		
Days to first adult emergence				
Species	7		8.29	0.040*
*S. alterniflora*		51.25 (3.53)		
*S. anglica*		47.58 (3.10)		
*S. maritime*		53.10 (1.93)		
*S. x townsendii*		50.25 (2.82)		

Mean values are given for each host species ± 1 S.E.M

Significance levels indicated by: * ≤ 0.05

**Fig. 4:**
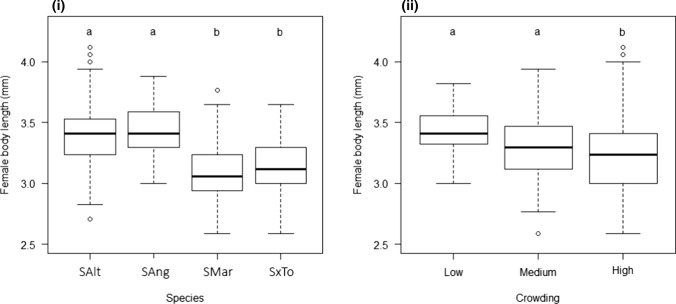
Effect of **i** host plant species and **ii** levels of crowding on body length of female *P. marginata.* Species abbreviations as per Fig. 1. Crowding levels: low (10 individuals added); Medium (30 individuals added); High (50 individuals added). Boxes show the interquartile range, the enclosed horizontal line representing the median. The tails of the vertical dashed lines represent approximately 2 standard deviations around the interquartile range in the presence of outliers (circles), or the full extent of the data where outliers are not present. Treatments sharing lower case letters are not significantly different from each other (Tukey HSD post-hoc test, using 95% CI)

Both host plant species (*χ*^2^ = 30.43, *p* < 0.001) and levels of crowding (*χ*^2^ = 12.07, *p* = 0.002) had a significant impact on the body size of female, but not male, *P. marginata*. The mean body length of females raised in the high crowding treatment was 6.4% lower than that of females raised in the low crowding treatment across all host species. The interaction between host plant species and crowding level was not significant (*χ*^2^ = 9.6, *p* = 0.143), however post-hoc tests showed that females reared on *S. alterniflora* and *S. anglica* were significantly larger than those reared on the other species (Fig. 4).

Feeding by *P. marginata* significantly reduced height and leaf length relative growth rates and relative above ground water content for all host species, but, with the exception of relative water content, the impact was least severe for *S. alterniflora*. *S. maritima* was most severely impacted by *P. marginata* exposure in terms of relative leaf length gain (reduced by 65% in comparison to control means) and relative water content (reduced by 70%), but not for relative height gain, for which *S. anglica* suffered the greatest comparative reduction (79%). Planthopper exposure also reduced the number of new leaves by as much as 66%. This metric did not vary significantly between plant species, however the impact was greatest for *S. maritima* and least severe for *S. alterniflora* (Table 2).

**Table 2:** Effect of *P. marginata* exposure, *Spartina* species and their interactions on plant performance measured as: height; total leaf length; and relative shoot water content

	d.f	Control (C) mean (SEM)	Treatment (T) mean (SEM)	T:C ratio	Test statistic	*p*
Relative height gain						
Treatment	1				58.74^a^	< 0.001***
Species	3				30.33^a^	< 0.001***
*S. alterniflora*		0.45 (0.07)	0.33 (0.05)	0.73		
*S. anglica*		0.28 (0.09)	0.06 (0.02)	0.21		
*S. maritime*		0.94 (0.08)	0.48 (0.07)	0.51		
*S. x townsendii*		0.59 (0.09)	0.14 (0.02)	0.23		
Treatment x species	3				3.08^a^	0.033*
Relative leaf length gain						
Treatment	1				100.88^a^	< 0.001***
Species	3				12.21^a^	< 0.001***
*S. alterniflora*		1.69 (0.11)	1.08 (0.09)	0.64		
*S. anglica*		1.16 (0.10)	0.60 (0.07)	0.51		
*S. maritime*		2.59 (0.30)	0.91 (0.11)	0.35		
*S. x townsendii*		1.96 (0.19)	0.89 (0.12)	0.45		
Treatment x species	3				4.31^a^	0.007**
Relative water content						
Treatment	1				303.24^a^	< 0.001***
Species	3				5.96^a^	0.001***
*S. alterniflora*		1.82 (0.04)	1.02 (0.11)	0.46		
*S. anglica*		1.63 (0.09)	0.89 (0.07)	0.55		
*S. maritime*		1.84 (0.07)	0.56 (0.03)	0.30		
*S. x townsendii*		1.61 (0.06)	0.58 (0.11)	0.36		
Treatment x species	3				4.85^a^	0.004**
No. new leaves gained						
Treatment	1				78.37^b^	< 0.001***
Species	3				6.27^b^	0.099
*S. alterniflora*		8.2 (0.55)	4.5 (0.82)	0.55		
*S. anglica*		7.5 (0.58)	2.7 (0.58)	0.36		
*S. maritime*		7.9 (0.84)	2.7 (0.50)	0.34		
*S. x townsendii*		9.3 (0.98)	4.1 (0.60)	0.44		
Treatment x species	3				3.44^b^	0.329
Residuals (all models)	72					

Relative metrics indicate ratios of post- to pre-experiment measurements. Mean values are given for each host species ± 1 S.E.M. Treatment refers to 30 *P. marginata* individuals added; Control refers to no *P. marginata*. Test statistics are (a) *F* values for two-way ANOVAs and (b) *χ*2 for GLMs

Significance levels indicated by: * ≤ 0.05; ** ≤ 0.01;*** ≤ 0.001

## Discussion

Previous studies have examined the impact of *P. marginata* herbivory only on *S. alterniflora*, its native host, as well as *S. anglica* populations that were introduced to Puget Sound, Washington, in 1961. *S. alterniflora* populations that had been separated from *P. marginata* for over 100 years and *S. anglica* populations with no prior experience of the planthopper suffered significant detrimental impacts when exposed to the herbivore (Daehler and Strong 1997; Wu et al. 1999; Garcia-Rossi et al. 2003; Grevstad et al. 2003). Our work extends investigation of the planthopper’s impacts to a unique species complex of native and introduced host species in the novel geographical context of Britain: an introduced species (*S. alterniflora*), a native species (*S. maritima*), a sterile homoploid hybrid between the two (*S*. x *townsendii*) and a fertile allododecaploid species arising from the last of these due to a chromosomal doubling event (*S. anglica*).

Our first prediction was that *P. marginata* would preferentially oviposit on *S. alterniflora.* Planthopper oviposition was found to be significantly affected by plant species, but results differed between experiments done under field compared to glasshouse conditions. As predicted, field planthopper populations showed a significant preference for *S. alterniflora* over *S. maritima*, although there were no other significant pairwise differences. Under controlled glasshouse conditions, *P. marginata* unexpectedly showed a significant preference for *S. anglica* over all other species. It should be noted, however, that all planthoppers used in the experiments were cultured on *S. anglica*, raising the possibility of prior conditioning to this host species (Coaker and Cheah 1993). However, as *S. anglica* is now the most abundant, widespread and dominant *Spartina* species in Britain, this is increasingly likely to reflect the reality of field conditions (Harkin and Stewart 2020).

Our second hypothesis predicted that *P. marginata* would achieve the greatest performance outcomes on *S. alterniflora*, the host to which it is expected to be best adapted by virtue of their shared evolutionary history, and on which it has previously been shown to achieve the greatest survival in no-choice host specificity tests (Denno et al. 1986; Grevstad et al. 2003). Empirical evidence from our no-choice host tests offers some support for this prediction; however, overall performance outcomes were again unexpectedly greatest on *S. anglica*, the host species most favoured by ovipositing females under controlled glasshouse conditions. Nymphal development was significantly faster for individuals raised on *S. anglica* than for those raised on *S. alterniflora* and *S. maritima,* and eggs laid on *S. anglica* hatched earlier than those laid on any other species (although differences here were not statistically significant). Faster development times are expected to confer fitness benefits because individuals more rapidly escape the elevated mortality risks associated with the vulnerable egg and nymph stages (Stiling and Strong 1982), whilst potentially increasing their lifetime realised fecundity as a consequence of achieving first reproduction earlier (Denno 1985). *P. marginata* eclose as larger adults when raised on nutritionally superior hosts (Denno et al. 1986) and there is a positive correlation between body size and the average daily fecundity of females (Denno and McCloud 1985). In the experiments reported here, females were significantly larger when raised on *S. alterniflora* and *S. anglica,* although host plant species did not appear to mitigate the significant negative effects of crowding on body size. Greater fecundity and survivorship were realised on *S. anglica* with significantly larger final populations achieved per host plant. In concert, the data provide evidence of *P. marginata* achieving relatively small, but significant, improvements in fitness-related performance when utilising *S. anglica* as its host plant. Further investigation is needed to determine the mechanisms underlying this outcome, however the apparently rapid pre-conditioning suggested by our oviposition choice tests indicates that *P. marginata* can quickly develop a preference for the most abundant suitable host and enhanced performance on it. It is possible therefore, that *P. marginata* has undergone, or is in the process of, rapid adaptation to maximise its performance on *S. anglica* in the 15–20 years since its arrival because *S. anglica* is the most abundant and widespread potential host species in Britain (Lacambra et al. 2004; Harkin and Stewart 2020). Further studies comparing British and North American populations of *P. marginata* would be instructive in testing this hypothesis.

The founding population of *S. alterniflora* in Britain can be considered to have shared a long, unbroken, evolutionary history with *P. marginata* prior to its arrival. This founding population would initially have had high levels of defence against the herbivore, as suggested by previous studies in North America (Daehler and Strong 1995; Gustafson et al. 2006), although this may subsequently have been eroded due to a lack of herbivore selection pressure in the ~ 200 years since its arrival. In contrast, *S. maritima* which is native to Europe had no exposure to *P. marginata* prior to the planthopper’s arrival and there are no known equivalent specialist *Spartina* herbivores native to Britain (Payne 1973). Therefore, *S. maritima* might be expected to display a lack of defence against *P. marginata* consistent with that reported for previously unexposed congeners (Wu et al. 1999).

Exposure to *P. marginata* had a significant negative impact on all four species of *Spartina* for all performance metrics, but subtle differences were evident in the severity of impacts between species. Three of the plant traits showed strong effects of exposure to planthopper feeding: relative height and leaf length growth rates, and relative water content. These traits reflect a composite metric of the cost of herbivory, plant compensatory growth and photosynthetic potential, indicative of differential impacts of *P. marginata* herbivory on the four species (Strauss and Agrawal 1999). Due to the sparse and extremely localised distribution of all but *S. anglica*, species identity and collection site are necessarily confounded in this study. However, we suggest the abiotic effects associated with collection site will have been minimised by the lengthy period of acclimation to glasshouse conditions prior to the start of experiments.

Our third hypothesis, that the impact of *P. marginata* herbivory would be least severe for *S. alterniflora*, was supported by the results. Even though British populations of the grass are likely to have been separated from the herbivore for ~ 200 years, *S. alterniflora* remained the least severely impacted of all four species examined. Additionally, we predicted that *S. maritima* would suffer the greatest detrimental impact of exposure to *P. marginata* herbivory and oviposition because it shares no evolutionary history with the planthopper, nor with any other specialist herbivore (Payne 1973). This prediction was only partially supported. *S. maritima* suffered significant negative impacts of exposure on all performance metrics, however the relative impact in comparison to the other *Spartina* species was variable, being the most severely impacted for some traits, but not for others. It is interesting to note that *S. maritima* plants in the control group (i.e. no herbivory) performed substantially better than the control group for any other species, displaying the greatest relative height and leaf length mean growth rates, and the highest relative shoot water content. In its current British distribution, *S. maritima* is extremely localised, sparsely populated and routinely out-competed by *S. anglica* (Lacambra et al. 2004); in our experiments, plants were grown individually in separate pots and hence freed from competition. *P. marginata* is currently in the early stages of invasion in the UK (Harkin and Stewart 2020). The results presented here suggest that its continued population growth and spread may pose a significant additional threat to the future survival of *S. maritima* across its remaining British distribution. Further experiments directly examining the comparative impacts of herbivory, competition and apparent competition on the interaction between *P. marginata*, *S. anglica* and *S. maritima* would be valuable to inform future management interventions.

We found no support for our final hypothesis: that the negative effect of *P. marginata* exposure would not differ between the allododecaploid *S. anglica* and its hexaploid progenitor *S. x townsendii*, as a result of their shared level of evolutionary history with the herbivore. Treatment means for all traits apart from relative height gain differed between these species, although further work would be needed to determine the role that ploidy levels may have played in this result. The differences may result from divergent evolutionary changes due to selective adaptation or random processes such as genetic drift (Schluter 2001). *S. anglica* was formed as a separate species ~ 130 years ago (Gray et al. 1991), but recent studies have shown that significant adaptive evolution can occur in a range of invasive plant species within twenty or fewer generations (Prentis et al. 2008). There is very little inter-individual genetic variation in *S. anglica* populations, consistent with a severe genetic bottleneck due to the unique event which resulted in the origin of the species (Baumel et al. 2001), and very little structural change has been observed in the genomes of either *S. x townsendii* or *S. anglica* (Baumel et al. 2002a). However, considerable epigenetic alteration (Salmon et al. 2005) as well as changes to the transcriptome (Chelaifa et al. 2010) arose from both the hybridization event that led to the formation of *S. x townsendii*, and in the subsequent chromosome doubling which resulted in *S. anglica*. These epigenetic and regulatory changes are thought to account for the high levels of phenotypic plasticity documented for *S. anglica* (Renny-Byfield et al. 2010), and may similarly offer an explanation for the different responses to *P. marginata* that we found in the two species.

Whilst polyploidy remains relatively understudied, it has the potential to significantly impact the diversity and outcomes of plant–herbivore interactions (Segraves and Anneberg 2016). In this context, the multi-ploidy *Spartina* species complex present in Britain represents an excellent model for the study of the impact of ploidy levels on the invasiveness of plant species. The allododecaploid *S. anglica* is of recent origin, the hexaploid progenitors are all still extant, and there is wide agreement on the likely timings of speciation events based on historical records (Gray et al. 1991). The recent imposition of *P. marginata* herbivory on these species, additionally facilitating the study of a diversity of novel and re-connected interactions, further adds to the research value of this system, providing a tractable model for the study of the impact of ploidy levels on the evolutionary ecology of plant–insect interactions, and of how the complexity of these interactions may influence the outcome of invasion dynamics.

## Data Availability

The data that support the findings of this study are available in the Figshare repository https://doi.org/10.25377/sussex.13034399

## References

[CR1] Ainouche ML, Fortune PM, Salmon A, Parisod C, Grandbastien MA, Fukunaga K, Ricou M, Misset MT (2009). Hybridization, polyploidy and invasion: lessons from *Spartina* (Poaceae). Biol Invasions.

[CR2] Badmin J (2013). Cordgrass planthopper *Prokelisia marginata* (van Duzee) (Hem: Delphacidae) in Devon. Br J Entomol Nat Hist.

[CR3] Bates, D., M. Maechler, and B. Bolker. 2012. lme4: Linear mixed-effects models using S4 classes. http://CRAN.R-project.org/package=lme4.

[CR4] Baumel A, Ainouche ML, Levasseur JE (2001). Molecular investigations in populations of *Spartina anglica* C.E. Hubbard (Poaceae) invading coastal Brittany (France). Mol Ecol.

[CR5] Baumel A, Ainouche M, Kalendar R, Schulman AH (2002). Retrotransposons and genomic stability in populations of the young allopolyploid species *Spartina anglica* CE Hubbard (Poaceae). Mol Biol Evol.

[CR6] Baumel A, Ainouche ML, Bayer RJ, Ainouche AK, Misset MT (2002). Molecular phylogeny of hybridizing species from the genus *Spartina* Schreb. (Poaceae). Mol Phylogenet Evol.

[CR7] Bezemer TM, Harvey JA, Cronin JT (2014). Response of native insect communities to invasive plants. Annu Rev Entomol.

[CR8] Bortolus A, Adam P, Adams JB, Ainouche ML, Ayres D, Bertness MD, Bouma TJ, Bruno JF, Cacador I, Carlton JT, Castillo JM, Costa CSB, Davy AJ, Deegan L, Duarte B, Figueroa E, Gerwein J, Gray AJ, Grosholz ED, Hacker SD, Hughes AR, Mateos-Naranjo E, Mendelssohn IA, Morris JT, Munoz-Rodriguez AF, Nieva FJJ, Levin LA, Li B, Liu WW, Pennings SC, Pickart A, Redondo-Gomez S, Richardson DM, Salmon A, Schwindt E, Silliman BR, Sotka EE, Stace C, Sytsma M, Temmerman S, Turner RE, Valiela I, Weinstein MP, Weis JS (2019). Supporting Spartina: Interdisciplinary perspective shows Spartina as a distinct solid genus. Ecology.

[CR9] Burghardt KT, Tallamy DW (2013). Plant origin asymmetrically impacts feeding guilds and life stages driving community structure of herbivorous arthropods. Divers Distrib.

[CR10] Callaway JC, Josselyn MN (1992). The introduction and spread of smooth cordgrass (*Spartina alterniflora*) in South San Francisco Bay. Estuaries.

[CR11] Chelaifa H, Monnier A, Ainouche M (2010). Transcriptomic changes following recent natural hybridization and allopolyploidy in the salt marsh species *Spartina x townsendi*i and *Spartina anglica* (Poaceae). New Phytol.

[CR12] Chun YJ, van Kleunen M, Dawson W (2010). The role of enemy release, tolerance and resistance in plant invasions: linking damage to performance. Ecol Lett.

[CR13] Coaker TH, Cheah CA (1993). Conditioning as a factor in parasitoid host-plant preference. Biocontrol Sci Tech.

[CR14] Committee JNC (2007). Second Report by the UK under Article 17 on the implementation of the Habitats Directive from January 2001 to December 2006.

[CR15] Daehler CC, Strong DR (1995). Impact of high herbivore densities on introduced smooth cordgrass, *Spartina alterniflora*, invading San Francisco Bay, California. Estuaries.

[CR16] Daehler CC, Strong DR (1997). Reduced herbivore resistance in introduced smooth cordgrass (*Spartina alterniflora*) after a century of herbivore-free growth. Oecologia.

[CR17] de Blauwe H (2011). Leafhopper bug *Prokelisia marginata* (Hemiptera: Delphacidae), an exotic species dependent on cord-grass *Spartina townsendii*, now found on the Belgian coast. Strandvlo.

[CR18] deJonge RB, Bourchier RS, Jones IM, Smith SM (2019). Predicting the outcome of potential novel associations: interactions between the invasive *Vincetoxicum rossicum* and native western *Chrysochus* beetles. Biol Invasions.

[CR19] Denno RF, Rankin MA (1985). Fitness, population dynamics and migration in plantoppers: the role of host plants. Migration: mechanisms and adaptive significance.

[CR20] Denno RF, McCloud ES (1985). Predicting fecundity from body size in the planthopper *Prokelisia marginata* (Homoptera, Delphacidae). Environ Entomol.

[CR21] Denno RF, Douglass LW, Jacobs D (1986). Effects of crowding and host plant nutrition on a wing-dimorphic planthopper. Ecology.

[CR22] Denno RF, Roderick GK, Peterson MA, Huberty AF, Dobel HG, Eubanks MD, Losey JE, Langellotto GA (1996). Habitat persistence underlies intraspecific variation in the dispersal strategies of planthoppers. Ecol Monogr.

[CR23] Denno RF, Peterson MA, Gratton C, Cheng JA, Langellotto GA, Huberty AF, Finke DL (2000). Feeding-induced changes in plant quality mediate interspecific competition between sap-feeding herbivores. Ecology.

[CR24] Fox J (2003). Effect displays in R for generalised linear models. J Stat Softw.

[CR25] Garcia-Rossi D, Rank N, Strong DR (2003). Potential for self-defeating biological control? Variation in herbivore vulnerability among invasive *Spartina* genotypes. Ecol Appl.

[CR26] Gray AJ, Marshall DF, Raybould AF (1991). A century of evolution in *Spartina anglica*. Adv Ecol Res.

[CR27] Gray, A. J., A. F. Raybould, and S. L. Brown. 1997. The environmental impact of *Spartina anglica*: past, present and predicted. Pages 39–45, Olympia, WA.

[CR28] Grevstad FS, Strong DR, Garcia-Rossi D, Switzer RW, Wecker MS (2003). Biological control of *Spartina alterniflora* in Willapa Bay, Washington using the planthopper *Prokelisia marginata*: agent specificity and early results. Biol Control.

[CR29] Gripenberg S, Mayhew PJ, Parnell M, Roslin T (2010). A meta-analysis of preference-performance relationships in phytophagous insects. Ecol Lett.

[CR30] Groves H, J. Groves (1880) *Spartina townsendii nobis* Report of the Botanical Society & Exchange Club of the British Isles **1**:37

[CR31] Gustafson DJ, Kilheffer J, Silliman BR (2006). Relative effects of *Littoraria irrorata* and *Prokelisia marginata* on *Spartina alterniflora*. Estuaries Coasts.

[CR32] Halverson K, Heard SB, Nason JD, Stireman JO (2008). Differential attack on diploid, tetraploid, and hexaploid *Solidago altissima* L. by five insect gallmakers. Oecologia.

[CR33] Harkin C, Stewart AJA (2020). Establishment, spread and impact of an invasive planthopper on its invasive host plant: *Prokelisia marginata* (Homoptera: Delphacidae) exploiting *Spartina anglica* (Poales: Poaceae) in Britain. Ecol Entomol.

[CR34] Hoagland DR, Arnon DI (1950). The water culture method for growing plants without soil. Calif Agric Exp Stn Circ.

[CR35] Hothorn T, Bretz F, Westfall P (2008). Simultaneous inference in general parametric models. Biom J.

[CR36] Hubbard CE, Hubbard JCE, Sampson J (1968). Grasses. 2nd edition.

[CR37] Hull-Sanders HM, Johnson RH, Owen HA, Meyer GA (2009). Influence of polyploidy on insect herbivores of native and invasive genotypes of Solidago gigantea (Asteraceae). Plant Signal Behav.

[CR38] IPBES (2019). Global assessment report on biodiversity and ecosystem services of the Intergovernmental Science-Policy Platform on Biodiversity and Ecosystem Services.

[CR39] Keane RM, Crawley MJ (2002). Exotic plant invasions and the enemy release hypothesis. Trends Ecol Evol.

[CR40] Lacambra C, Cutts N, Allen J, Burd F, Elliott M (2004). English Nature Research Reports No. 527 Spartina anglica: a review of its status, dynamics and management.

[CR41] Lou Y, Baldwin IT (2003). *Manduca sexta* recognition and resistance among allopolyploid *Nicotiana* host plants. Proc Natl Acad Sci USA.

[CR42] Lu X, Ding J (2012). History of exposure to herbivores increases the compensatory ability of an invasive plant. Biol Invasions.

[CR43] Mack RN, Simberloff D, Lonsdale WM, Evans H, Clout M, Bazzaz FA (2000). Biotic invasions: causes, epidemiology, global consequences, and control. Ecol Appl.

[CR44] Marchant CJ (1967). Evolution in *Spartina* (Gramineae): I. The history and morphology of the genus in Britain. J Linn Soc London, Bot.

[CR45] Maron JL, Vila M (2001). When do herbivores affect plant invasion? Evidence for the natural enemies and biotic resistance hypotheses. Oikos.

[CR46] Mitchell CE, Agrawal AA, Bever JD, Gilbert GS, Hufbauer RA, Klironomos JN, Maron JL, Morris WF, Parker IM, Power AG, Seabloom EW, Torchin ME, Vazquez DP (2006). Biotic interactions and plant invasions. Ecol Lett.

[CR47] Mooney HA, Cleland EE (2001). The evolutionary impact of invasive species. Proc Natl Acad Sci USA.

[CR48] Munzbergova Z (2006). Ploidy level interacts with population size and habitat conditions to determine the degree of herbivory damage in plant populations. Oikos.

[CR49] Munzbergova Z, Skuhrovec J, Marsik P (2015). Large differences in the composition of herbivore communities and seed damage in diploid and autotetraploid plant species. Biol J Lin Soc.

[CR50] Pandit MK, Pocock MJO, Kunin WE (2011). Ploidy influences rarity and invasiveness in plants. J Ecol.

[CR51] Pandit MK, White SM, Pocock MJO (2014). The contrasting effects of genome size, chromosome number and ploidy level on plant invasiveness: a global analysis. New Phytol.

[CR52] Payne K (1973). A survey of the *Spartina*-feeding insects in Pool Harbour, Dorset. Entomol Monthly Mag.

[CR53] Pearse IS, Harris DJ, Karban R, Sih A (2013). Predicting novel herbivore-plant interactions. Oikos.

[CR54] Pinheiro, J., D. Bates, S. DebRoy, D. Sarker, and R Development Core Team. 2012. nlme: Linear and Nonlinear Mixed Effects Models. http://CRAN.R-project.org/web/packages/nlme/citation.html.

[CR55] Prentis PJ, Wilson JRU, Dormontt EE, Richardson DM, Lowe AJ (2008). Adaptive evolution in invasive species. Trends Plant Sci.

[CR56] R Core Team (2015). R: A language and environment for statistical computing.

[CR57] Ramsey J, Schemske DW (1998). Pathways, mechanisms, and rates of polyploid formation in flowering plants. Annu Rev Ecol Syst.

[CR58] Raybould AF, Gray AJ, Lawrence MJ, Marshall DF (1991). The evolution of *Spartina anglica* CE Hubbard (Gramineae) - origin and genetic variability. Biol J Lin Soc.

[CR59] Renny-Byfield S, Ainouche M, Leitch IJ, Lim KY, Le Comber SC, Leitch AR (2010). Flow cytometry and GISH reveal mixed ploidy populations and *Spartina* nonaploids with genomes of *S. alterniflora* and *S. maritima* origin. Ann Bot.

[CR60] Roberts PD, Pullin AS (2008). The effectiveness of management interventions for the control of *Spartina* species: a systematic review and meta-analysis. Aquat Conservation-Marine Freshw Ecosyst.

[CR61] Salmon A, Ainouche ML, Wendel JF (2005). Genetic and epigenetic consequences of recent hybridization and polyploidy in *Spartina* (Poaceae). Mol Ecol.

[CR62] Schluter D (2001). Ecology and the origin of species. Trends Ecol Evol.

[CR63] Segraves KA, Anneberg TJ (2016). Species interactions and plant polyploidy. Am J Bot.

[CR64] Seljak G (2004). *Prokelisia marginata* (Van Duzee, 1897) - a Nearctic planthopper, new to Slovenia and Europe (Auchenorrhyncha: Delphacidae). Acta Entomologica Slovenica.

[CR65] Soltis PS, Soltis DE (2000). The role of genetic and genomic attributes in the success of polyploids. Proc Natl Acad Sci USA.

[CR66] Stebbins GL (1956). Cytogenetics and evolution of the grass family. Am J Bot.

[CR67] Stiling PD, Strong DR (1982). Egg density and the intensity of parasitism in Prokelisia marginata (Homoptera, Delphacidae). Ecology.

[CR68] Strauss SY, Agrawal AA (1999). The ecology and evolution of plant tolerance to herbivory. Trends Ecol Evol.

[CR69] Strong DR, Ayres DR (2013). Ecological and evolutionary misadventures of *Spartina*. Annu Rev Ecol Evol Syst.

[CR70] te Beest M, Le Roux JJ, Richardson DM, Brysting AK, Suda J, Kubesova M, Pysek P (2012). The more the better? The role of polyploidy in facilitating plant invasions. Ann Bot.

[CR71] Thompson JD (1991). The biology of an invasive plant: What makes *Spartina anglica* so successful?. Bioscience.

[CR72] Thompson JN, Nuismer SL, Merg K (2004). Plant polyploidy and the evolutionary ecology of plant/animal interactions. Biol J Lin Soc.

[CR73] Wu MY, Hacker S, Ayres D, Strong DR (1999). Potential of *Prokelisia* spp. as biological control agents of English cordgrass *Spartina anglica*. Biol Control.

[CR74] Zangerl AR, Berenbaum MR (2005). Increase in toxicity of an invasive weed after reassociation with its coevolved herbivore. Proc Natl Acad Sci USA.

